# Supplementary material for: Utilization of social media communities for caregiver information support in stroke recovery: An analysis of content and interactions
Source: PLoS One. 2022 Jan 26;17(1):e0262919. doi: 10.1371/journal.pone.0262919 (PMC8791510; doi:10.1371/journal.pone.0262919)
Supplement: Attachment — Submitted filename: Journal and Reviewers Comments.docx [file pone.0262919.s004.docx]

**Journal and Reviewers Comments**

| **Journal Requirements** | | |
| --- | --- | --- |
| Comment | Response | Action |
| Please ensure that your manuscript meets PLOS ONE's style requirements, including those for file naming. | Thank you for your feedback. | We have made the following changes based on the PLOS One Style Requirements:   - Title page   - Updated the title to be in Sentence Case and centre aligned   - Updated the affiliation information   - Updated the corresponding author information to the author email address followed by the author initials within a parentheses. - Manuscript Body   - Removed the title   - Tab spacing for new paragraphs   - Replaced **Figure X** with **Fig X**   - Added figure caption directly after each paragraph which they are cited   - Used bold types for figures and tables captions   - Added Supporting Information for minimal dataset |
| Thank you for stating the following in the Acknowledgments Section of your manuscript:  “This study was supported through doctoral scholarships from the School of Information Technology, Deakin University, and the Department of Public Health, University of Copenhagen. Further, Prof John Grundy is supported by ARC Laureate Fellowship FL190100035, and Dr. Sarah Hosking is supported by an Alfred Deakin Postdoctoral Research Fellowship.”  We note that you have provided additional information within the Acknowledgements Section that is not currently declared in your Funding Statement. Please note that funding information should not appear in the Acknowledgments section or other areas of your manuscript. We will only publish funding information present in the Funding Statement section of the online submission form.  Please remove any funding-related text from the manuscript and let us know how you would like to update your Funding Statement. Currently, your Funding Statement reads as follows:  “The author(s) received no specific funding for this work.”  Please include your amended statements within your cover letter; we will change the online submission form on your behalf. | Thank you for your feedback. We have removed the Acknowledgements section from the manuscript body, and would like to move that entire paragraph into the funding statement. | Please replace the funding statement text with the text below:  This study was supported through doctoral scholarships from the School of Information Technology, Deakin University, and the Department of Public Health, University of Copenhagen. Further, Prof John Grundy is supported by ARC Laureate Fellowship FL190100035, and Dr. Sarah Hosking is supported by an Alfred Deakin Postdoctoral Research Fellowship |
| In your Data Availability statement, you have not specified where the minimal data set underlying the results described in your manuscript can be found. PLOS defines a study's minimal data set as the underlying data used to reach the conclusions drawn in the manuscript and any additional data required to replicate the reported study findings in their entirety. All PLOS journals require that the minimal data set be made fully available. For more information about our data policy, please see http://journals.plos.org/plosone/s/data-availability.  Upon re-submitting your revised manuscript, please upload your study’s minimal underlying data set as either Supporting Information files or to a stable, public repository and include the relevant URLs, DOIs, or accession numbers within your revised cover letter. For a list of acceptable repositories, please see http://journals.plos.org/plosone/s/data-availability#loc-recommended-repositories. Any potentially identifying patient information must be fully anonymized. | Thank you for your feedback. We have included the Minimal Data Set including the communities included along with the meta-data, and the various types of posts included that provide information support. This is included in the supporting information section of the manuscript. |  |
| **Reviewer 1** | | |
| The manuscript has been nicely constructed, addressing a very relevant social aspect in stroke care. One suggestion is to add to the discussion part, is the possibility of gathering misinformation from less trusted sources in the internet and it's possible I'll effects also. | Thank you for your feedback. We have made the necessary changes to address this suggestion. | ***On Pages 18-19 Lines 266-277, we have added:***  Nowadays, misinformation or lack of quality information is a growing problem [24]. Crocco, Villasis-Keever [25] in a systematic review highlighted the internet’s capacity to harm the health of the user to be equal to the good and useful information it provides in a relatively timely and inexpensive manner. For example, in one case the misinformation available on the internet contributed to emotional harm, while in another case lead to hepatorenal failure in an oncology patient who obtained misinformation regarding medication use over the internet [25]. To prevent healthcare issues and fears amongst the population, Cuan-Baltazar, Muñoz-Perez [24] suggests the need for governmental organizations to develop a strategy that teaches its residents to verify the quality of information they read. Moreover, Swire-Thompson and Lazer [26] describes the need for internet users to collaborate with physicians to ensure they are more actively involved in the decision-making processes, and they are aware of methods to separate health myths from facts that the internet provides. |
| **Reviewer 2** | | |
| Please describe the web application tool which was developed to extract information from all social media. Whether there is any process to test the reliability of the tool? How to make sure that the tool has extracted the data as the researchers intended to do and whether it was consistent across social media platforms? | Thank you for your feedback. The web application tool developed included a form where the researcher would manually input information from the social media page. This was not an automated process. To make it clearer, we have updated the text to ensure the reader is aware of the process. | ***Previously Pages 5-6 Lines 86-89 read:***  Initially, a web application tool was developed to extract information from all social media communities, including community names, descriptions, links, number of followers (or likes), and several posts, and store the data in a MySQL database. The communities identified were then filtered based on the inclusion and exclusion criteria described in Table 1.  ***Now it reads:***  Initially, **the researchers used a custom-built web form to manually extract information** from all social media communities, including community names, descriptions, links, number of followers (or likes), and several posts, and then store this data in a MySQL database. The communities identified were then filtered based on the inclusion and exclusion criteria described in Table 1. |
| The authors have described the research approach as a mixed method approach. If you explain the research design adopted, it would be beneficial to the readers. | Thank you for your feedback. We have updated the text to reflect this change. | ***Previously Page 4-5 Lines 60-69 read:***  Our study consisted of a mixed-method approach to answer identified research aims. For example, to investigate the frequency of stroke terms, a quantitative analysis was conducted to determine the online activity of people interested in stroke recovery and care using tools such as Google Trends and Google Insights. Google Trends and Google Insights provide a platform for individuals to investigate its users' search behaviour throughout time based on a relative cumulative search volume score from 0-100, which is the ratio of single search term volume to all possible searches. A qualitative analysis was used to analyse the information content using a thematic synthesis approach. Finally, the levels of interaction were identified through a quantitative statistical analysis of likes, comments, and shares based on the different content types.  ***Now it reads:***  Our study consisted of a mixed-method approach to answer identified research aims. **The mixed methods approach is a type of research where a researcher or group of researchers combine elements of quantitative and qualitative methods (e.g. use of quantitative and qualitative viewpoints for data collection, analysis and inference techniques) to provide a broad understanding of the research problem [21].** For example, to investigate the frequency of stroke terms, a quantitative analysis was conducted to determine the online activity of people interested in stroke recovery and care using tools such as Google Trends and Google Insights. Google Trends and Google Insights provide a platform for individuals to investigate its users' search behaviour throughout time based on a relative cumulative search volume score from 0-100, which is the ratio of single search term volume to all possible searches. A qualitative analysis was used to analyse the information content using a thematic synthesis approach. Finally, the levels of interaction were identified through a quantitative statistical analysis of likes, comments, and shares based on the different content types. |
| Any report on geographical location of the data could be identified? | Thank you for your question. The data was collected worldwide, and hence we did not discuss the geographical location. However, we have made some changes to the text to make it clearer. | ***Previously Page 5 Lines 74-76 read:***  We tested the keywords on Google Insights and Google Trends to determine their relevance to the individual in stroke recovery and care based on their searching behaviour.  ***Now it reads:***  We tested the keywords on Google Insights and Google Trends to determine their relevance **to individuals around the world** in stroke recovery and care based on their searching behaviour |
| Some of the keywords are pure medical terms and could have been searched by students of medicine, nursing or allied health professions. Is there a way to say it with certainty that the searches were made by caregivers? Common man may use terms like paralysis, weakness, not able to talk etc. for stroke. | Thank you for your feedback. There is no certain way of identifying if the terms used were made by caregivers, which is why we haven’t discussed the relevance of keywords with respect to caregivers in the Google Trends and Insights search process. The goal of this stage is to show the increase in online use of people interested in stroke recovery and care (Lines 127-138), and identify the terms that they might use to search for online communities (Lines 144-147).  The keywords in Table 2 represent just the top 10, the remaining keywords are available in the **Supporting Information File S1**. All these terms were identified through topic experts and past online searches as already described in the text Lines 73-74. |  |
| “The average 139 rise in the cumulative search volume was 12.4 from January 2011 to December 2020” Please explain how this value is calculated and what is the unit of the value. Explain the numbers on the ‘X’ axis of figure 1. | Thank you for your feedback.  We have updated the text to reflect how it was calculated.  The cumulative search volume has no unit of value, and hence was not included.  We have included labels for Figure 1 to represent X axis as the years over 10 years period and Y axis as the cumulative search volume for each search category. | ***Previously Lines 134-135 read:***  The average rise in the cumulative search volume was 12.4 from January 2011 to December 2020  ***Now it reads:***  The rise in the cumulative search volume was 12.4 between January 2011 and December 2020 identified by:  $a_{ij}= \frac{\sum k_{i}}{N_{ij}} (1)$  Where $a_{ij}$ is the average cumulative search volume for each topic ($j$) each year ($i$), $k$ is the cumulative search volume acquired from Google Trends and Insights for all the keywords associated with the topic for year $i$, $N$ is the total number of keywords in the topic ($j$) for year $i$, $i$ is the year ranging from 1 to 10 and $j$ is the topic ranging from 1 to 4.  $Y_{i}= \frac{\sum_{j=1}^{j=4} a_{ij}}{4} (2)$  where $Y_{i}$ is the average cumulative search volume for all topics ($a_{ij}$) in year $i$ ranging from 1 to 10  $A= Y_{10}- Y_{1} (3)$  where $A$ is the rise in the cumulative search volume between the Tenth ($Y_{10}$) and First ($Y_{1}$) Years (i.e. January 2011 and December 2020) |
| Please explain how ‘Traumatic Brain Injury’ can be a keyword to identify stroke? | Thank you for your query. We found ‘Traumatic Brain Injury’ (TBI) as a keyword often used with stroke in Google Trends. While TBI and stroke are different, TBI can be an exposure factor for stroke [1,2] and might be difficult to interpret by common people using social media platforms.  [1] Burke JF, Stulc JL, Skolarus LE, Sears ED, Zahuranec DB, Morgenstern LB. Traumatic brain injury may be an independent risk factor for stroke. Neurology. 2013 Jul 2;81(1):33-9.  [2] Meschia JF. Traumatic brain injury and stroke. In Mayo Clinic Proceedings 2014 Feb 1 (Vol. 89, No. 2, pp. 142-143). Elsevier. |  |
| In the exclusion criteria it was mentioned that sites used for Promotion of Products and Services were excluded whereas in the analysis 14 sites are included whose purpose was advertising | Thank you for letting us know. We have deleted that criteria from **Table 1**.  The exclusion of products and services was supposed to be for the social media posts and not the communities. As most communities might describe themselves as an advertising community but have information resources regarding the benefits of a particular tool in the daily lives of the caregiver, which is relevant to this study. |  |
| 64 (32.3%)sites were created by individuals. Whether this report could bring out the authenticity of the content on those sites? | Thank you for the query. While we identified the number of sites, determining the authenticity was out of scope and not intended. However, we have described the issues related to misinformation and its impact on the individual On Pages 18-19 Lines 266-277 based on Reviewer #1 feedback. | ***On Pages 18-19 Lines 266-277, we have added:***  Nowadays, misinformation or lack of quality information is a growing problem [24]. Crocco, Villasis-Keever [25] in a systematic review highlighted the internet’s capacity to harm the health of the user to be equal to the good and useful information it provides in a relatively timely and inexpensive manner. For example, in one case the misinformation available on the internet contributed to emotional harm, while in another case lead to hepatorenal failure in an oncology patient who obtained misinformation regarding medication use over the internet [25]. To prevent healthcare issues and fears amongst the population, Cuan-Baltazar, Muñoz-Perez [24] suggests the need for governmental organizations to develop a strategy that teaches its residents to verify the quality of information they read. Moreover, Swire-Thompson and Lazer [26] describes the need for internet users to collaborate with physicians to ensure they are more actively involved in the decision-making processes, and they are aware of methods to separate health myths from facts that the internet provides. |
| The content of table 5 has to be rechecked. ‘what is stroke’ query in Facebook by the reviewer generated more than one videos and plenty of likes, shares and comments. Other areas also need to be checked. | Thank you for your query. Table 5 provides an average value for likes, shares and comments for different content types (explained in Lines 240-241). As most of the videos have very little interaction for ‘What is Stroke’ the average value is low.  To make it clearer to the reader, we have updated the caption to let the reader know that these values are averages. | ***Previously Table 5 caption read:***  Table 5 Analysis of User Interaction  ***Now it reads:***  Table 5 Analysis of User Interaction based on the Averages of Likes, Shares and Comments for different Content Types |
